# Missense mutations at homologous positions in the fourth and fifth laminin A G**-**like domains of eyes shut homolog cause autosomal recessive retinitis pigmentosa

**Published:** 2010-12-15

**Authors:** Muhammad Imran Khan, Rob W.J. Collin, Kentar Arimadyo, Shazia Micheal, Maleeha Azam, Nadeem Qureshi, Sultana M.H. Faradz, Anneke I. den Hollander, Raheel Qamar, Frans P.M. Cremers

**Affiliations:** 1 Department of Biosciences, COMSATS Institute of Information Technology, Islamabad, Pakistan; 2 Department of Human Genetics, Radboud University Nijmegen Medical Centre, Nijmegen, The Netherlands; 3 Department of Ophthalmology, Radboud University Nijmegen Medical Centre, Nijmegen, The Netherlands; 4 Nijmegen Centre for Molecular Life Sciences, Radboud University Nijmegen Medical Centre, Nijmegen, The Netherlands; 5 Division of Human Genetics, Center for Biomedical Research, Faculty of Medicine, Diponegoro University, Semarang, Indonesia; 6 Vitreoretina Services, Al-Shifa Trust Eye Hospital, Rawalpindi, Pakistan; 7 Shifa College of Medicine, Islamabad, Pakistan

## Abstract

**Purpose:**

To describe two novel mutations in the eyes shut homolog (*EYS*) gene in two families with autosomal recessive retinitis pigmentosa (arRP) from Pakistan and Indonesia.

**Methods:**

Genome-wide linkage and homozygosity mapping were performed using single nucleotide polymorphism microarray analysis in affected members of the two arRP families. Sequence analysis was performed to identify genetic changes in protein coding exons of *EYS.*

**Results:**

In the Indonesian and Pakistani families, homozygous regions encompassing the *EYS* gene at 6q12 were identified, with maximum LOD scores of 1.8 and 3.6, respectively. Novel missense variants in the *EYS* gene (p.D2767Y and p.D3028Y) were found in the Pakistani and Indonesian families, respectively, that co-segregate with the disease phenotype. Interestingly, the missense variants are located at the same homologous position within the fourth and fifth laminin A G-like domains of EYS.

**Conclusions:**

To date, mostly protein-truncating mutations have been described in *EYS,* while only few patients have been described with pathogenic compound heterozygous missense mutations. The mutations p.D2767Y and p.D3028Y described in this study affect highly conserved residues at homologous positions in laminin A G-like domains and support the notion that missense mutations in *EYS* can cause arRP.

## Introduction

The term retinitis pigmentosa (RP) describes a clinically diverse and genetically heterogeneous group of progressive hereditary degenerative disorders with a worldwide prevalence of approximately 1 in 4,000 individuals [1–3]. RP is characterized by nyctalopia, progressive mid-peripheral visual field loss, and at later stages involvement of the macula and complete loss of visual acuity [4]. The clinical diagnosis of RP relies upon the bilateral presence of intraretinal pigment deposits (commonly known as bone spicules), attenuation of retinal vessels, atrophy of the peripheral retinal pigmented epithelium, and waxy pallor of the optic disc [4].

RP is familial in about 60% of the cases and can present all documented forms of inheritance [3]. To date, 45 causative genes and nine loci have been identified (RetNet). Twenty-seven genes have been identified for the autosomal recessive form of RP (arRP), which together are believed to account for approximately 50% of all cases [5]. Most of these genes account for 1%–2% of arRP cases, while ATP-binding cassette, sub-family A, member 4 (*ABCA4;* OMIM 601691*)*, eyes shut homolog (*EYS*; OMIM 612424), and Usher syndrome type 2A (*USH2A*; OMIM 608400) seem to be more frequently mutated in arRP patients [2,6–8].

Eyes shut homolog (*EYS*; OMIM 612424), a gene that is expressed specifically in the retina, was implicated in the pathology of arRP 2 years ago [7,8]. Recent reports suggest a global involvement of *EYS*, with a high prevalence of protein-truncating mutations in this gene [9–12]. In the present study, we report two families from Pakistan and Indonesia that harbor homozygous missense mutations in *EYS*.

## Methods

### Patients and clinical evaluation

**Figure 1:**
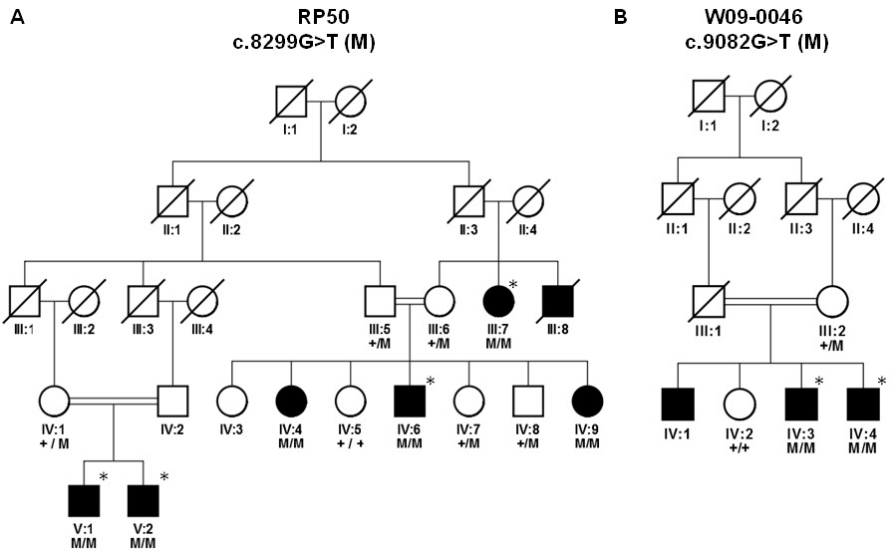
Pedigree structure and segregation analysis of mutations in families. **A**: Family RP50; M represents c.8299G>T. **B**: Family W09–0046; M represents c.9082G>T. Genotypes are indicated below the genetic symbols. Asterisks indicate the individuals that were analyzed on single nucleotide polymorphism arrays.

The present study comprises two consanguineous families with arRP. Family RP50 (Figure 1A) was collected from the Punjab region of Pakistan, while family W09–0046 (Figure 1B) was from the Java island of Indonesia. This study was approved by the institutional review boards of the respective institutions and was conducted in accordance with the Declaration of Helsinki.

The initial diagnostic criterion used for the recruitment of patients was the presence of night blindness. One affected proband and one normal individual from each family was selected for detailed clinical examination, including funduscopy and electroretinography (ERG). ERG recordings were performed in accordance with the guidelines provided by the International Society for Clinical Electrophysiology of Vision, using a monopolar contact lens electrode (LKC Inc., Gaithersburg, MD) [13].

### Genetic studies

Peripheral blood was drawn by venipuncture, and stored at −20 °C till further processing. DNA was extracted using a standard phenol-chloroform extraction protocol [14]. Genomic DNA of four affected individuals from family RP50 (Figure 1A) and two affected individuals from family W09–0046 (Figure 1B) were analyzed using Illumina 6K single nucleotide polymorphism (SNP) arrays that contain 6,090 SNP markers (Illumina., Inc., San Diego, CA). To identify regions that might harbor the genetic defects in the two families, linkage analysis was performed using the Gene Hunter program (version 2.1r5) in the easyLinkage plus software package (version 5.08) [15].

One affected individual from each family was selected for mutation analysis. All 44 exons and the flanking intronic sequences of *EYS* were amplified using PCR, with primers described previously [7]. The amplicons were subsequently analyzed by sequencing using dye termination chemistry (BigDye Terminator, version 1.1 on a 3730 DNA analyzer; Applied Biosystems Inc., Foster City, CA). Segregation analysis was performed for the variants identified by sequence analysis. Restriction fragment length polymorphism (RFLP) analysis and amplification refractory mutation detection system (ARMS)-PCR were performed in controls and additional arRP patients to analyze the presence of sequence variants identified in this study. For the mutation detected in family RP50, a 510-bp product containing exon 44 was amplified for restriction digestion, using primers ex44F: 5′-CAC AAT TGT GCT CAA GAT CTG-3′ and ex44R: 5′-TAC ATT TGA GCC ACC TTT TGC-3′. Restriction digestion with HphI yields two fragments of 130 and 380 bp for the wild-type allele, while the mutant allele remains undigested. For the mutation in family W09–0046, an ARMS-PCR was performed to screen 90 ethnically matched controls, using the wild-type specific primer 5′-GGA TGG GAA TAG CTC AAA ATG AAG AAA ACG-3′ or the mutant allele specific 5′-GGA TGG GAA TAG CTC AAA ATG AAG AAA ACT-3′ primer, together with the common reverse primer 5'-GTT TAG AGC CAC AAA GTT TTT ATG TGG ATC-3′.

Various web-based bioinformatic tools were used to assess the pathogenic potential of the missense variants identified in this study, including project Hope, Polymorphism Phenotyping (PolyPhen), and Sorting Intolerant From Tolerant (SIFT). To assess the evolutionary conservation, amino acid sequences of orthologous EYS proteins (XP_001918194, XP_001505792.1, XP_426198.2, NP_001027571.2) and of the homologous human protein laminin α1 (LAMA1, NP_005550) were obtained from the NCBI protein database and were aligned with the protein sequence of human EYS, using Vector NTI software (version 11, Invitrogen, Carlsbad, CA).

## Results

**Figure 2:**
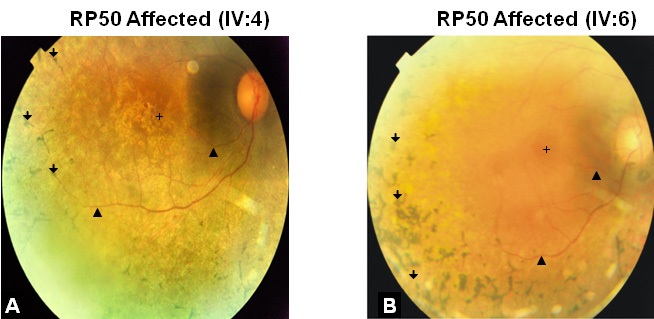
Fundus photographs of autosomal recessive retinitis pigmentosa patients of family RP50. Fundus photographs of the right eye from affected individual IV:4 (**A**) and affected individual IV:6 (**B**) are shown. Ages at the time of investigation of individuals IV:4 and IV:6 were 40 and 38, respectively. Examination of the fundi reveals bone spicule pigmentations at the periphery (arrows), and attenuated retinal vessels (arrowheads). The crosses indicate the center of the maculae which are degenerated in both cases.

Family RP50 (Figure 1A) consists of five generations with seven affected individuals, one of whom was deceased. All patients presented with night blindness that started early in the second decade of life. Fundus examinations revealed bone spicules at the retinal periphery and attenuation of retinal vasculature (Figure 2A,B). The scotopic ERG, which represents the rod response, is more diminished compared to the photopic ERG, indicating a typical rod–cone dystrophy (Table 1). The low visual acuity (VA) in these patients is suggestive of macular involvement. The macular degeneration is vigorously progressive, as indicated by significant loss of VA in less than 2 years (Table 2).

**Table 1:** Electroretinography (ERG) response comparison between affected and normal individual of family RP50.

**ERG measurements**	**Adaptation**	**Flash strength**	**IV:6**	**IV:8**	Normal values*
Scotopic 25 dB b-wave amplitude (µV)	Dark	0.01 (cds/m2)	12.6	175.5	>141
Scotopic 0 dB b-wave amplitude (µV)	Dark	3.0 (cds/m2)	38.6	469.4	>387
Oscillatory potential amplitude (µV)	Dark	3.0 (cds/m2)	133.1	284.9	>75
Photopic 0 dB b-wave amplitude (µV)	Light	3.0 (cds/m2)	32.4	156.7	>82
Photopic 30 Hz flicker amplitude (µV)	Light	3.0 (cds/m2)	28.5	92.1	>56

Electrical responses of eyes to different intensities of light are compared to discriminate between the rod and cone response. The scotopic ERG, which represents the rod response, is more diminished in patient IV:6 compared to the photopic ERG, indicating a typical rod-cone dystrophy in family RP50.*Normal values at 30–40 years of age. Age at the time of investigation of individual IV:6 (affected) and IV:8 (normal) were 38 and 32, respectively.

**Table 2:** Clinical characteristics of families RP50 and W09–0046.

					**VA at current age**		**VA at the time of diagnosis**	
**Family**	**Patient**	**Current age**	**Gender**	**Age of Onset**	**RE (OD)**	**LE (OS)**	**RE (OD)**	**LE (OS)**
RP50	IV:4	42	Female	12–15	20/630	20/630	20/400	20/400
	IV:6	40	Male	12–15	20/125	20/400	20/125	20/125
W09–0046	IV:3	48	Male	16–18	ND	ND	20/400	20/400
	IV:4	42	Male	16–18	ND	ND	20/400	20/400

The table indicates the loss of visual acuity which points toward the macular degeneration. For family RP50, the comparison between the readings taken at an earlier and later time points, point toward the rapid progression of the disease. Current age is indicated in the table while the age at the time of diagnosis for individuals IV:4 and IV:6 from RP50 were 40 and 38 years and for IV:3 and IV:4 from family W09–0046, were 46 and 40, respectively. Abbreviations: VA, uncorrected visual acuity; ND, not determined; RE, right eye; LE, left eye.

Family W09–0046 (Figure 1B) consists of four generations with two affected and two healthy individuals that were available for genotyping. Onset of night blindness was at adolescence, whereas fundus examination revealed bone spicules and arterial attenuation (no fundus picture available). All patients had a low visual acuity.

Genome-wide linkage analysis in four affected individuals (III:7, IV:6, V:1, V:2) from family RP50 revealed only one region with a significant LOD score of 3.6. The region spans 25.2 Mb between rs283545 and rs2998367 and encompasses the *EYS* gene (Table 3). A similar analysis in two affected individuals (IV:3, IV:4) from family W09–0046 revealed three regions with a maximum LOD score of 1.8 (Table 3). The largest of these homozygous regions (69.5 Mb; flanked by rs2763122 and rs1486039) also encompasses the *EYS* gene.

**Table 3:** Linkage analysis of families RP50 and W09–0046

**Family**	**Chromosome**	**LOD**	**Flanking SNPs**	**hg18 position (Mb)**
RP50	6	3.6	rs283545 ; rs2998367	51,07–76,27
	10	3.1	rs2394829 ; rs703990	73,01–80,60
W09–0046	5	1.8	rs444984 ; rs1030154	154,43 – 164,98
	6	1.8	rs2763122 ; rs1486039	7,42- 76,96
	16	1.8	rs741175 ; rs2925508	11,07–18,77

Multipoint linkage analysis results are shown, i.e., logarithm of the odds (Lod) scores for each chromosomal region, the SNPs flanking the homozygous regions, as well as their physical positions in the hg18 NCBI genome assembly. For family RP50 the region on chromosome 6 with the LOD score 3.6 is the largest (25.2 Mb) homozygous region. For family W09–0046 three regions show equally high (1.8) LOD scores, the largest of which (69.5 Mb) is also located on chromosome 6.

**Figure 3:**
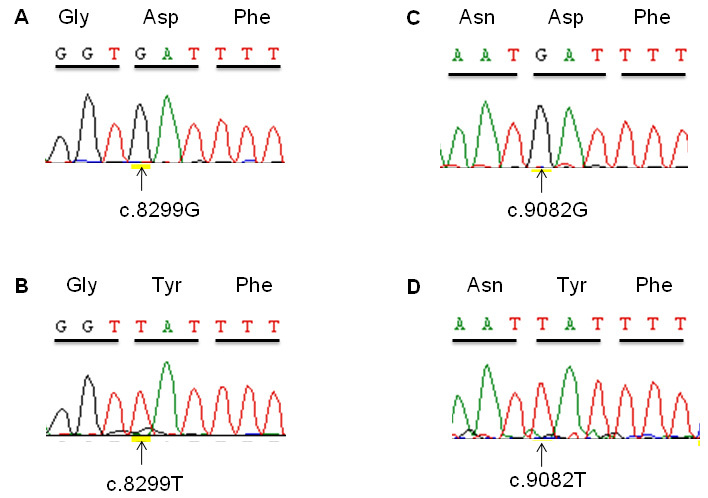
Eyes shut homolog (*EYS*) mutation analysis. Part of exon 44 sequence chromatograms are shown for (**A**) unaffected individual IV:5 from family RP50, (**B**) affected individual IV:6 from family RP50, (**C**) unaffected individual IV:2 from family W09–0046, and (**D**) affected individual IV:3 from family W09–0046. The encoded amino acids are indicated above the respective codons. The substituted nucleotides are indicated by arrows.

Sequence analysis of all 44 exons and flanking intronic regions of *EYS* revealed novel homozygous missense mutations—p.D2767Y (c.8299G>T) and p.D3028Y (c.9082G>T)—in the probands of families RP50 and W09–0046, respectively (Figure 3). Sequence analysis in all relatives available for genotyping indicated that the two missense mutations segregate with the disease in the respective families (Figure 1). Missense mutation p.D2767Y was not found in a panel of 81 Pakistani probands affected with RP. Likewise, this variant was not found in 90 ethnically matched control individuals from Pakistan. ARMS analysis for the missense mutation p.D3028Y in 90 controls from the Indonesian population also yielded no carrier of the mutation.

### In silico analysis

**Figure 4:**
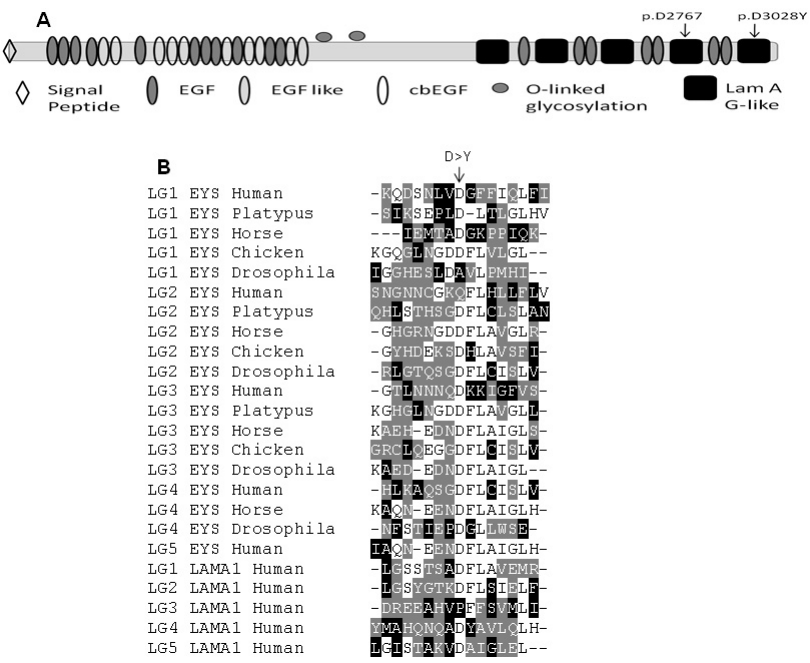
Domain architecture and amino acid sequence comparison of eyes shut homolog (EYS), orthologs and human laminin α1 (LAMA1) laminin G domains. **A**: Domain architecture of human EYS is shown, indicating the position of mutations in the functional domains. Variants p.D2767Y and p.D3028Y are located in the fourth and fifth laminin A G-like domains, respectively. **B**: Sequence comparison of laminin A G-like domains of human EYS with its orthologs and human LAMA1. The arrow indicates the position of the mutated aspartic acid residue in the alignment.

Intriguingly, at the protein level the aspartic acid (D) residues at positions 2,767 and 3,028 that are mutated in the Pakistani and Indonesian family are located at homologous positions in the fourth and fifth laminin A G-like domain of EYS (Figure 4A). Both missense changes result in the substitution of a tyrosine residue (Y) for an aspartic acid (D). The wild-type aspartic acid, being negatively charged and hydrophilic, might be involved in the ionic interactions at the surface, whereas the mutant tyrosine is an uncharged and less hydrophilic residue. As such, the missense changes might abolish ionic interactions or alter protein conformation. In an alignment of laminin A G-like domains of five EYS orthologous proteins, the aspartic acid residues D2,767 and D3,028 in the fourth and fifth laminin A G-like (Figure 4B) domains of human EYS are conserved in 17 of 19 homologous sequences. In addition, in most of the laminin G (LG) domains from the human LAMA1 protein an aspartic acid residue is present at the same position within these domains (Figure 4B). These data suggest that the presence of an aspartic acid residue at that position in the laminin A G-like domains is important for the proper function of these domains.

## Discussion

In the present study we describe two homozygous missense mutations in the *EYS* gene, which has recently been identified as the mutated gene at the RP25 locus at 6p12.1-q15 [7,8]. The RP25 locus was designated to be a major locus for arRP as approximately 20% of the Spanish arRP families and many other families were linked to this locus [16]. Numerous *EYS* mutations have recently been identified in approximately 5%–10% of arRP patients from different ethnic backgrounds [7,8,10–12]. *EYS* spans 2 Mb of genomic DNA, encodes 3,165 amino acids and to date is the largest gene identified to be specifically expressed in the retina. Immunolocalization studies in the adult pig retina revealed that EYS is present only in the outer photoreceptor cell layer where it is confined to the rod outer segments along with rhodopsin [8]. The function of EYS in the human retina is not yet established but its ortholog in *Drosophila*, known as eyes shut or spacemaker, is involved in luminal space formation that optically isolates neighboring rhabdomers from photoreceptor cells in the compound eye and thus enables each photoreceptor to act as an independent optical unit [7,8,17]. In *Drosophila*, the absence of EYS functionally results in “photoreceptor fusion,” a structure that resembles the “primitive” ommatidium in most insects, like ants and bees [17,18]. To form the open rhabdomer configuration, EYS needs to interact with Prominin 1 (Prom1; OMIM 604365), another protein implicated in arRP, and absence of any of these proteins results in a closed rhabdomer formation [18]. In a *Prom1*^−/−^ mouse model, the absence of Prom1 leads to progressive degeneration of cone and rod photoreceptors due to impaired morphogenesis of the photoreceptor disks. It is therefore thought that human EYS is also involved in correct morphogenesis of photoreceptors [18–20].

EYS consists of 21 epidermal growth factor (EGF)-like domains at the N-terminus, followed by five carboxy terminal laminin A G-like domains with alternating EGF-like domains [7,8]. The conserved domain database reveals that in EYS orthologs, the laminin A G-like domain architecture is well conserved, although the different orthologous EYS proteins have variable numbers of these domains. Given the similarities in sequence and domain architecture of EYS compared to laminin proteins, we speculate that in vivo EYS might attain a structure similar to the LG domain complex. Laminin-1, one of the two basic components of basement membranes, forms a cruciform structure, with the α, β, and γ laminin chains, arranged in heterotrimeric geometry. The α-chain of laminin-1 forms an extended structure and, like EYS, carries five globular domains at its C-terminus, termed LG domains. These LG domains form a sandwich structure where LG1–LG3 attain a clover leaf arrangement, which is connected through a small linker to the C-terminal LG domain pair formed by LG4 and LG5, with LG4 being positioned at the distal end [21,22]. This LG domain complex in laminin forms the receptor-binding site, where LG1–LG3 binds integrin, while the LG4–LG5 pair binds the transmembrane glycoprotein dystroglycan. Considering this and the in silico prediction of the mutations we identified in the fourth and fifth LG domains of EYS, our data suggest that these mutations might impair the interaction of EYS with putative partners.

The novel missense mutations identified here are conspicuously located at the same homologous position in laminin A G-like domains of EYS, while most of the previously identified pathogenic mutations are protein truncating [7,8,11]. Recently, compound heterozygous missense mutations (p.C2139Y and p.G2186E) have been described in a Chinese family, while another missense variant (p.R589G) was found in conjunction with a protein-truncating mutation (p.Y2555X) in a sporadic RP patient [10]. The variant p.C2139Y resides in the twenty-first EGF-like domain of EYS, while p.G2186E is located in the second laminin A G-like domain. The alignment of the laminin A G-like domains indicates that p.G2186 is not fully conserved in all the other laminin domains, neither of EYS nor of laminin α (data not shown). Audo et al. described novel *EYS* variants in 29 families but only in one family two missense mutations were identified [12]. These mutations are not located in a functional domain of EYS. Several other missense variants were found heterozygously and not conclusively considered pathogenic or were not shown to segregate via different alleles [12]. The p.D2767Y and p.D3028Y mutations in our study are the first homozygous missense variants that independently show pathogenic potential. Based on the maximal LOD score of 1.8, however, we cannot be certain that the *EYS* variant c.9082G>T (p.D3028Y) in family W09–0046 is causative.

Taken together, our data not only reveal the importance of the laminin A G-like domains in the function of the EYS protein but also the critical role of the mutated residue aspartic acid for laminin A G-like domains. In addition, the identification of novel *EYS* mutations in the Pakistani and Indonesian population further supports a global involvement of *EYS* mutations as a cause of arRP.
